# Extinction Analysis of Stochastic Predator–Prey System with Stage Structure and Crowley–Martin Functional Response

**DOI:** 10.3390/e21030252

**Published:** 2019-03-06

**Authors:** Conghui Xu, Guojian Ren, Yongguang Yu

**Affiliations:** School of Science, Beijing Jiaotong University, Beijing 100044, China

**Keywords:** predator–prey system, stochastically ultimate boundedness, stochastic extinction, Brownian motion, Crowley–Martin functional response

## Abstract

In this paper, we researched some dynamical behaviors of a stochastic predator–prey system, which is considered under the combination of Crowley–Martin functional response and stage structure. First, we obtained the existence and uniqueness of the global positive solution of the system. Then, we studied the stochastically ultimate boundedness of the solution. Furthermore, we established two sufficient conditions, which are separately given to ensure the stochastic extinction of the prey and predator populations. In the end, we carried out the numerical simulations to explain some cases.

## 1. Introduction

Population dynamics is one of the main parts of biological mathematics. The predator–prey model is a classical problem in population research. Lotka and Volterra [1] researched the origin and theory of predator–prey model, which is given by

(1) $$\begin{array}{c} \left\{\begin{array}{c} \frac{dx}{dt}=a_{0}x\left(t\right)-c_{0}x\left(t\right)y\left(t\right),\\ \frac{dy}{dt}=e_{0}c_{0}x\left(t\right)y\left(t\right)-d_{0}y\left(t\right), \end{array}\right. \end{array}$$

where x(t) and y(t) represent the population density of the prey and predator, respectively; $a_{0}$ and $d_{0}$ denote the intrinsic growth rate and death rate, respectively; and $c_{0}$ and $e_{0}$ are the predation rate of a predator and nutrient-conversion rate, respectively.

An important feature of the predator–prey relationship is the functional response (i.e., the rate of prey consumption by an average predator). Mukherjee [2] discussed persistence and bifurcation on the predator–prey system of Holling Type II. Liu and Zhong [3] researched permanence and extinction for the delayed periodic predator–prey system with Holling Type II response function and diffusion. Zhang and Yang [4] studied Hopf bifurcation in the predator–prey system with Holling Type III functional response and time delays. The functional responses of Holling Types I–III are prey-dependent, which have been researched by many scholars. However, the functional response is inevitably influenced by the behavior of a predator, such as foraging and competing. Therefore, many scholars studied various types of predator-dependent functions. Gilliam and Skalski [5] claimed that the predator-dependent can provide better descriptions of predators feeding over a range of predator–prey abundances by comparing the statistical evidence from 19 predator–prey systems with the three predator-dependent functional responses (Hassell–Varley [6], Beddington–DeAngelis [7,8,9], and Crowley–Martin [10,11]), and, in some cases, the Crowley–Martin functional response is better. On the other hand, compared with the Hassell–Varley and Beddington–DeAngelis functional responses, the Crowley–Martin functional response is more suitable for the case that the predator feeding rate is decreased by higher predator density even when prey density is high. Thus, we consider the Crowley–Martin functional response in this paper.

In the classical predator–prey model, it is always assumed that each predator has the same predation capacity, and each prey has the same risk of predation. This assumption is unrealistic for many species. In nature, there are many species whose individuals have a life history that takes them through two stages, immature and mature. Individuals of different age groups exhibit different biological behaviors. In view of this, many scholars have studied the predator–prey system with stage structure [12,13,14,15,16]. Sun and Huo [17] considered bifurcation and stability in the predator–prey model with stage structure for the predator. Xu [18] discussed the global dynamics of the predator–prey model with time delay and stage structure for the prey. Lu [19] studied the stage-structured predator–prey model with predation over juvenile prey. However, few researchers have studied the predator–prey model with a stage structure for predator and prey. In nature, we know that immature predators have no predatory capacity. Meanwhile, many species hatch from eggs. For example, the Saltcedar leaf beetle is such a pest. In view of its eggshell, pathogens may not be effective against an immature pest. Based on this situation, it is reasonable to assume that immature prey does not run the risk of being preyed on. In terms of pest and disease control, the stage-structure model can better describe the dynamic behavior of some species. Therefore, in this paper, we mainly consider the predator–prey system with stage structure for both predator and prey.

We also consider the impact of environmental noise. Many scholars have studied various types of stochastic predator–prey systems with stage structure and functional-response functions [20,21,22]. Liu and Jiang [23] researched the dynamics of a stochastic predator–prey model with stage structure for predator and Holling Type II functional response. Chen and You [24] studied permanence, extinction, and periodic solution of the predator–prey system with a Beddington–DeAngelis functional response and stage structure for prey. Liu and Zhong [25] discussed the asymptotic properties of a stochastic predator–prey model with a Crowley–Martin functional response.

The main contributions of our work can be summarized as follows. The predator–prey model with random perturbation and Crowley–Martin functional response is established, which consider stage structure on both prey and predator. The existence and uniqueness of the global positive solution of the system is proved. Some sufficient conditions are given, which ensure the solutions of the system are stochastically ultimate boundedness. Then, sufficient conditions for the extinction of prey and predator are given, respectively. Finally, the conclusion is verified by numerical simulation results.

The paper is organized as follows. In Section 2, we give two prey–predator models with stage structure and a Crowley–Martin functional response. One is deterministic, and another is stochastic, which is discussed through the manuscript. In Section 3, we prove the existence and uniqueness of the global positive solution. In Section 4, we obtain sufficient conditions for stochastically ultimate boundedness of the prey and predator. In Section 5, we establish sufficient conditions for extinction of the predator and prey in two cases. The first case is the prey and predator extinction; another case is the predator extinction. In Section 6, numerical simulations illustrate the theoretical results. Section 7 gives the conclusions and future research directions.

## 2. Preliminaries

Before giving the main results, we first introduce some mathematical symbols and formulas in this paper. Throughout this paper, we define

$$R_{+}^{q}=\left\{x=\left(x_{1},x_{2},\cdots ,x_{q}\right)\in R^{q}:x_{i}>0,1\leq i\leq q\right\}.$$

Consider the process of *q*-dimensional $It\hat{o}$

(2) $$\begin{array}{c} dX(t)=LV(X(t),t)dt+g(X(t),t)dB(t), \end{array}$$

where $B\left(t\right)=(B_{1}\left(t\right),B_{2}\left(t\right),\cdots ,B_{q}\left(t\right)$) denotes independent standard Brownian motions defined on a complete probability space $\left(\Omega ,F,{\left\{F_{t}\right\}}_{t\geq 0},P\right)$ with a filtration ${\left\{F_{t}\right\}}_{t\geq 0}$, and assume that the constant initial value $X_{0}\in R^{q}$. Differential operator *L* of Formula (4) is given by

$$L=\frac{\partial }{\partial t}+\sum_{i=1}^{q}f_{i}\left(X\left(t\right),t\right)\frac{\partial }{\partial X_{i}}+\frac{1}{2}\sum_{i,j=1}^{q}{\left[g\left(X\left(t\right),t\right),g{\left(X\left(t\right),t\right)}^{T}\right]}_{ij}\frac{\partial^{2}}{\partial x_{i}\partial x_{j}}.$$

We denote a function V(x(t),t) defined on $C^{2,1}\left(R^{q},R\right)$. Applying *L* on V(X(t),t), one has

$$LV\left(X\left(t\right),t\right)=V_{t}\left(X\left(t\right),t\right)+V_{X}\left(X\left(t\right),t\right)f\left(X\left(t\right),t\right)+\frac{1}{2}trace\left[g^{T}\left(X\left(t\right),t\right)V_{XX}\left(X\left(t\right),t\right)g\left(X\left(t\right),t\right)\right],$$

where $V_{t}=\frac{\partial V}{\partial t}$, $V_{X}=\left(\frac{\partial V}{\partial X_{1}},\frac{\partial V}{\partial X_{2}},\cdots ,\frac{\partial V}{\partial X_{q}}\right)$, $V_{XX}={\left(\frac{\partial^{2}V}{\partial X_{i}\partial X_{j}}\right)}_{d\times d}$. By $It\hat{o}$ formula, we can obtain

$$dV\left(X\left(t\right),t\right)=LV\left(X\left(t\right),t\right)dt+V_{X}\left(X\left(t\right),t\right)g\left(X\left(t\right),t\right)dB\left(t\right).$$

If the system is autonomous, the definition of operator *L* and $It\hat{o}$ formula discussed above can be found in Reference [26].

Based on the statement in Section 1, consider the following model:

(3) $$\begin{array}{c} \left\{\begin{array}{c} \frac{dx_{1}\left(t\right)}{dt}=ax_{2}\left(t\right)-d_{1}x_{1}\left(t\right)-px_{1}\left(t\right),\\ \frac{dx_{2}\left(t\right)}{dt}=px_{1}\left(t\right)-d_{2}x_{2}\left(t\right)-b_{1}x_{2}^{2}\left(t\right)-\frac{cx_{2}\left(t\right)y_{2}\left(t\right)}{1+\alpha x_{2}\left(t\right)+\beta y_{2}\left(t\right)+\alpha \beta x_{2}\left(t\right)y_{2}\left(t\right)},\\ \frac{dy_{1}\left(t\right)}{dt}=\frac{ecx_{2}\left(t\right)y_{2}\left(t\right)}{1+\alpha x_{2}\left(t\right)+\beta y_{2}\left(t\right)+\alpha \beta x_{2}\left(t\right)y_{2}\left(t\right)}-d_{3}y_{1}\left(t\right)-hy_{1}\left(t\right),\\ \frac{dy_{2}\left(t\right)}{dt}=hy_{1}\left(t\right)-d_{4}y_{2}\left(t\right)-b_{2}y_{2}^{2}\left(t\right), \end{array}\right. \end{array}$$

where $x_{1}\left(t\right)$ and $x_{2}\left(t\right)$ denote the densities of immature and mature prey at time *t*, respectively; $y_{1}\left(t\right)$ and $y_{2}\left(t\right)$ represent the densities of immature and mature predators at time *t*, respectively; the parameters *a*, $d_{1}$, $d_{2}$, $d_{3}$, $d_{4}$, $b_{1}$, $b_{2}$, *p*, *h*, *e* and *c* are positive constants, *a* is the birth rate of immature prey, *p* and *h* indicate maturity rate of immature prey and immature predator, respectively; $b_{1}$ and $b_{2}$ express the competition rate between a mature prey population and mature predator population, respectively; $d_{1}$ and $d_{2}$ are the death rates of immature and mature prey, respectively; and $d_{3}$ and $d_{4}$ represent the death rates of immature and mature predators, respectively.

May [27] pointed out that due to continuous fluctuation in the environment, the birth rate, death rates, carrying capacity, competition coefficients, and all other parameters involved with the model exhibit random fluctuation. Thus, we consider environmental random disturbance as follows:

$$-d_{1}\to -d_{1}+\sigma_{1}\dot{B_{1}}\left(t\right),-d_{2}\to -d_{2}+\sigma_{2}\dot{B_{2}}\left(t\right),-d_{3}\to -d_{3}+\sigma_{3}\dot{B_{3}}\left(t\right),-d_{4}\to -d_{4}+\sigma_{4}\dot{B_{4}}\left(t\right),$$

where $B_{i}\left(t\right)\left(i=1,2,3,4\right)$ represent independent standard Brownian motions, and $\sigma_{i}\left(i=1,2,3,4\right)$ are the intensities of the environmental random disturbance. Then, we can obtain the following system:

(4) $$\begin{array}{c} \left\{\begin{array}{c} dx_{1}\left(t\right)=\left[ax_{2}\left(t\right)-d_{1}x_{1}\left(t\right)-px_{1}\left(t\right)\right]dt+\sigma_{1}x_{1}\left(t\right)dB_{1}\left(t\right),\\ dx_{2}\left(t\right)=\left[px_{1}\left(t\right)-d_{2}x_{2}\left(t\right)-b_{1}x_{2}^{2}\left(t\right)-\frac{cx_{2}\left(t\right)y_{2}\left(t\right)}{1+\alpha x_{2}\left(t\right)+\beta y_{2}\left(t\right)+\alpha \beta x_{2}\left(t\right)y_{2}\left(t\right)}\right]dt+\sigma_{2}x_{2}\left(t\right)dB_{2}\left(t\right),\\ dy_{1}\left(t\right)=\left[\frac{ecx_{2}\left(t\right)y_{2}\left(t\right)}{1+\alpha x_{2}\left(t\right)+\beta y_{2}\left(t\right)+\alpha \beta x_{2}\left(t\right)y_{2}\left(t\right)}-d_{3}y_{1}\left(t\right)-hy_{1}\left(t\right)\right]dt+\sigma_{3}y_{1}\left(t\right)dB_{3}\left(t\right),\\ dy_{2}\left(t\right)=\left[hy_{1}\left(t\right)-d_{4}y_{2}\left(t\right)-b_{2}y_{2}^{2}\left(t\right)\right]dt+\sigma_{4}y_{2}\left(t\right)dB_{4}\left(t\right). \end{array}\right. \end{array}$$

In this paper, we mainly research some population characteristics of System (3).

## 3. Existence and Uniqueness of Global Positive Solution

As we know, the density of population $x_{1}\left(t\right)$, $x_{2}\left(t\right)$, $y_{1}\left(t\right)$ and $y_{2}\left(t\right)$ should be positive. Therefore, we give the following theorem to ensure that the system has a unique positive solution.

**Theorem** **1.** *For any given initial data $x_{1}\left(0\right)>0$, $x_{2}\left(0\right)>0$, $y_{1}\left(0\right)>0$ and $y_{2}\left(0\right)>0$, there is a unique solution $\left(x_{1}\left(t\right),x_{2}\left(t\right),y_{1}\left(t\right),y_{2}\left(t\right)\right)$ to System (3), and the solution remains in $R_{+}^{4}$ with probability 1.*

**Proof of Theorem** **1.** Since System (3) satisfies the local Lipschitz continuous condition, there is a local unique solution $\left\{x_{1}\left(t\right),x_{2}\left(t\right),y_{1}\left(t\right),y_{2}\left(t\right)\right\}\in R_{+}^{4}$ for any initial data $\left\{x_{1}\left(0\right),x_{2}\left(0\right),y_{1}\left(0\right),y_{2}\left(0\right)\right\}\in R_{+}^{4}$ on $t\in [0,\tau_{e})$ (with probability 1), where $\tau_{e}$ is the explosion time. To show that the solution is global, we only need to prove $\tau_{e}=\infty$ a.s. Give the following conditions for the initial value:

$$\frac{1}{l_{0}}<min\left\{x_{1}\left(0\right),x_{2}\left(0\right),y_{1}\left(0\right),y_{2}\left(0\right)\right\}\leq max\left\{x_{1}\left(0\right),x_{2}\left(0\right),y_{1}\left(0\right),y_{2}\left(0\right)\right\}<l_{0},$$

where $l_{0}$ is a sufficiently large number. For each integer $l\geq l_{0}$, define the stopping time

$$\tau_{l}=inf\left\{t\in \left(0,\tau_{e}\right):x_{1}\left(t\right)\notin \left(\frac{1}{l},l\right)\;or\;x_{2}\left(t\right)\notin \left(\frac{1}{l},l\right)\;or\;y_{1}\left(t\right)\notin \left(\frac{1}{l},l\right)\;or\;y_{2}\left(t\right)\notin \left(\frac{1}{l},l\right)\right\},$$

where, in this paper, we set inf∅=∞. According to the definition of $\tau_{l}$, it is clear that $\tau_{l}$ increases as l→∞. Set $\tau_{\infty }=lim_{l\to \infty }\tau_{l}$, whence $\tau_{\infty }\leq \tau_{e}$. That is to say, in order to prove the solution is global, it is sufficient to show that $\tau_{\infty }=\infty$ a.s. Then, we define a $C^{2}$-function *V*: $R_{+}^{4}\to R_{+}$ by

(5) $$\begin{array}{c} V\left(x_{1}\left(t\right),x_{2}\left(t\right),y_{1}\left(t\right),y_{2}\left(t\right)\right)=\sum_{i=1}^{2}\left[x_{i}\left(t\right)-1-\ln x_{i}\left(t\right)\right]+\sum_{i=1}^{2}\left[y_{i}\left(t\right)-1-\ln y_{i}\left(t\right)\right]. \end{array}$$

The non-negative of this function can be seen from

u−1−lnu≥0,∀u>0.

Let T>0, for $0\leq t\leq \tau_{m}\wedge T$. Applying It$\hat{o}$’s formula to $V(x_{1}\left(t\right),x_{2}\left(t\right),y_{1}\left(t\right),y_{2}\left(t\right))$, we have

(6) $$\begin{array}{c} d\left(V\left(x_{1},x_{2},y_{1},y_{2}\right)\right)=L\left(V\left(x_{1},x_{2},y_{1},y_{2}\right)\right)dt+\sum_{i=1}^{4}\left[\left(x_{i}-1\right)\sigma_{i}dB_{i}\left(t\right)\right]. \end{array}$$

According to the definition of operator *L*, we have

$$L\left(V\left(x_{1},x_{2},y_{1},y_{2}\right)\right)=\left[F_{1}\left(x_{1},x_{2},y_{1},y_{2}\right)+F_{2}\left(x_{1},x_{2},y_{1},y_{2}\right)+H_{1}\left(x_{1},x_{2},y_{1},y_{2}\right)+H_{2}\left(x_{1},x_{2},y_{1},y_{2}\right)\right]dt,$$

where $F_{1}\left(x_{1},x_{2},y_{1},y_{2}\right)=\left(1-\frac{1}{x_{1}}\right)\left(ax_{2}-d_{1}x_{1}-px_{1}\right)+\frac{\sigma_{1}^{2}}{2}$, $F_{2}\left(x_{1},x_{2},y_{1},y_{2}\right)=\left(1-\frac{1}{x_{2}}\right)\left(px_{1}-d_{2}x_{2}-b_{1}x_{2}^{2}-\frac{cx_{2}y_{2}}{1+\alpha x_{2}+\beta y_{2}+\alpha \beta x_{2}y_{2}}\right)+\frac{\sigma_{2}^{2}}{2}$, $H_{1}\left(x_{1},x_{2},y_{1},y_{2}\right)=\left(1-\frac{1}{y_{1}}\right)\left(\frac{ecx_{2}y_{2}}{1+\alpha x_{2}+\beta y_{2}+\alpha \beta x_{2}y_{2}}-d_{3}y_{1}-hy_{1}\right)+\frac{\sigma_{3}^{2}}{2}$ and $H_{2}\left(x_{1},x_{2},y_{1},y_{2}\right)=\left(1-\frac{1}{y_{2}}\right)\left(hy_{1}-d_{4}y_{2}-b_{2}y_{2}^{2}\right)+\frac{\sigma_{4}^{2}}{2}$.

Then, we have

(7) $$\begin{array}{c} \begin{array}{cc} LV(x_{1},x_{2},y_{1},y_{2}) & \leq -b_{1}x_{2}^{2}+\left(a+b_{1}\right)x_{2}-b_{2}y_{2}^{2}+b_{2}y_{2}+p+h+\frac{c}{\beta }+\frac{ec}{\alpha \beta }+\sum_{i=1}^{4}d_{i}+\sum_{i=1}^{4}\frac{\sigma_{i}^{2}}{2}\leq K, \end{array} \end{array}$$

where $K=\frac{{\left(a+b_{1}\right)}^{2}}{4b_{1}}+\frac{b_{2}}{4}+p+h+\frac{c}{\beta }+\frac{ec}{\alpha \beta }+\sum_{i=1}^{4}d_{i}+\sum_{i=1}^{4}\frac{\sigma_{i}^{2}}{2}>0$. It can be obtained from Formulas (6) and (7) that

(8) $$\begin{array}{c} d\left(V\left(x_{1},x_{2},y_{1},y_{2}\right)\right)\leq Kdt+\sum_{i=1}^{4}\left[\left(x_{i}-1\right)\sigma_{i}dB_{i}\left(t\right)\right]. \end{array}$$

Integrating both sides of Formula (8) from 0 to $\tau_{l}\wedge T$, we have

(9) $$\begin{array}{cc} V(x_{1}\left(\tau_{l}\wedge T\right),x_{2}\left(\tau_{l}\wedge T\right), & y_{1}\left(\tau_{l}\wedge T\right),y_{2}\left(\tau_{l}\wedge T\right))\leq V\left(x_{1}\left(0\right),x_{2}\left(0\right),y_{1}\left(0\right),y_{2}\left(0\right)\right)+KT\\ & +\int_{0}^{\tau_{l}\wedge T}\sigma_{1}\left(x_{1}-1\right)dB_{1}\left(t\right)+\int_{0}^{\tau_{l}\wedge T}\sigma_{2}\left(x_{2}-1\right)dB_{2}\left(t\right)\\ & +\int_{0}^{\tau_{l}\wedge T}\sigma_{3}\left(y_{1}-1\right)dB_{3}\left(t\right)+\int_{0}^{\tau_{l}\wedge T}\sigma_{4}\left(y_{2}-1\right)dB_{4}\left(t\right). \end{array}$$

Taking expectations at both sides of Formula (9), it is easy to obtain

(10) $$\begin{array}{c} \begin{array}{cc} EV(x_{1}\left(\tau_{l}\wedge T\right),x_{2}\left(\tau_{l}\wedge T\right),y_{1}\left(\tau_{l}\wedge T\right),y_{2}\left(\tau_{l}\wedge T\right)) & \leq M, \end{array} \end{array}$$

where $M=V(x_{1}\left(0\right),x_{2}\left(0\right),y_{1}\left(0\right),y_{2}\left(0\right))+KT$. According to the definition of $\tau_{l}$, there is some *i* (i=1,2), such that $x_{i}\left(\tau_{l},\omega \right)$ and $y_{i}\left(\tau_{l},\omega \right)$ equal either $\frac{1}{l}$ or *l*. Then, $V(x_{1}\left(\tau_{l},\omega \right),x_{2}\left(\tau_{l},\omega \right),y_{1}\left(\tau_{l},\omega \right),y_{2}\left(\tau_{l},\omega \right))$ is no less than either

$$l-1-\ln l\;\;\;or\;\;\;\frac{1}{l}-1-\ln\frac{1}{l}.$$

Then, one has

$$V\left(x_{1}\left(\tau_{l},\omega \right),x_{2}\left(\tau_{l},\omega \right),y_{1}\left(\tau_{l},\omega \right),y_{2}\left(\tau_{l},\omega \right)\right)\geq \left[\left(l-1-\ln l\right)\wedge \left(\frac{1}{l}-1-\ln\frac{1}{l}\right)\right].$$

According to Formula (10), we can obtain

(11) $$\begin{array}{c} \begin{array}{cc} M & \geq EV(x_{1}\left(\tau_{l\wedge T}\right),x_{2}\left(\tau_{l\wedge T}\right),y_{1}\left(\tau_{l\wedge T}\right),y_{2}\left(\tau_{l\wedge T}\right))\\ & \geq E[1_{\tau_{l}\leq T}\left(\omega \right)V\left(x_{1}\left(\tau_{l}\right),x_{2}\left(\tau_{l}\right),y_{1}\left(\tau_{l}\right),y_{2}\left(\tau_{l}\right)\right)]\\ & \geq P\left\{\tau_{l}\leq T\right\}\left[\left(l-1-\ln l\right)\wedge \left(\frac{1}{l}-1-\ln\frac{1}{l}\right)\right]. \end{array} \end{array}$$

Letting l→∞, we have

$$\lim_{l\to \infty }P\left\{\tau_{l}\leq T\right\}=0.$$

Since T>0 is arbitrary, we have

$$P\{\tau_{\infty }<\infty \}=0.$$

Then,

$$P\{\tau_{\infty }=\infty \}=1.$$

The proof of Theorem 1 is completed. □

## 4. Stochastically Ultimate Boundedness

Theorem 1 shows that the solution of System (3) remains in the positive cone $R_{+}^{4}$. However, this nonexplosion property in a population dynamical system is often not good enough. Therefore, the property of ultimate boundedness is more desired. First, we give the definition of stochastically ultimate boundedness.

**Definition** **1** ([28])**.** *With respect to System (3), the solution is said to be stochastically ultimate bounded, if for ϵ∈(0,1), there is a positive constant H=H(ϵ) such that for any initial data $\left\{x_{1}\left(0\right),x_{2}\left(0\right),y_{1}\left(0\right),y_{2}\left(0\right)\right\}\in R_{+}^{4}$, the solution $\left\{x_{1}\left(t\right),x_{2}\left(t\right),y_{1}\left(t\right),y_{2}\left(t\right)\right\}$ has the property that*

(12) $$\begin{array}{c} \underset{t\to \infty }{lim sup}P\left\{|X\left(t\right)|\geq H\right\}\leq \epsilon , \end{array}$$

*where $\left|X\left(t\right)\right|={\left(x_{1}^{2}+x_{2}^{2}+y_{1}^{2}+y_{2}^{2}\right)}^{\frac{1}{2}}$.*

**Assumption** **1.** *$\sigma_{1}^{2}-2d_{1}-2p+1<0$, $\sigma_{2}^{2}-2d_{2}+1<0$, $\sigma_{3}^{2}-2d_{3}-2h+1<0$ and $\sigma_{4}^{2}-2d_{4}+1<0$.*

**Theorem** **2.** *Under Assumption 1, the solution of System (3) is stochastically ultimately bounded for any initial data $\left\{x_{1}\left(0\right),x_{2}\left(0\right),y_{1}\left(0\right),y_{2}\left(0\right)\right\}\in R_{+}^{4}$.*

**Proof of Theorem** **2.** For $\left\{x_{1}\left(t\right),x_{2}\left(t\right),y_{1}\left(t\right),y_{2}\left(t\right)\right\}\in R_{+}^{4}$, define $V(x_{1}\left(t\right),x_{2}\left(t\right),y_{1}\left(t\right),y_{2}\left(t\right))$ as the following

$$V\left(x_{1}\left(t\right),x_{2}\left(t\right),y_{1}\left(t\right),y_{2}\left(t\right)\right)=\sum_{i=1}^{2}x_{i}^{2}\left(t\right)+\sum_{i=1}^{2}y_{i}^{2}\left(t\right).$$

By $It\hat{o}$’s formula, we have

(13) $$\begin{array}{c} dV\left(x_{1},x_{2},y_{1},y_{2}\right)=LV\left(x_{1},x_{2},y_{1},y_{2}\right)dt+2\sigma_{1}x_{1}^{2}dB_{1}\left(t\right)+2\sigma_{2}x_{2}^{2}dB_{2}\left(t\right)+2\sigma_{3}y_{1}^{2}dB_{3}\left(t\right)+2\sigma_{4}y_{2}^{2}dB_{4}\left(t\right). \end{array}$$

Therefore, it is easy to derive

(14) $$\begin{array}{cc} LV(x_{1},x_{2},y_{1},y_{2}) & =-2b_{1}x_{2}^{3}-2b_{2}y_{2}^{3}+\left(\sigma_{1}^{2}-2d_{1}-2p\right)x_{1}^{2}+\left(\sigma_{2}^{2}-2d_{2}\right)x_{2}^{2}+\left(\sigma_{3}^{2}-2d_{3}-2h\right)y_{1}^{2}\\ & \;+\left(\sigma_{4}^{2}-2d_{4}\right)y_{2}^{2}+2\left(a+p\right)x_{1}x_{2}+2hy_{1}y_{2}+\frac{2ecx_{2}y_{1}y_{2}}{1+\alpha x_{2}+\beta y_{2}+\alpha \beta x_{2}y_{2}}\\ & \;-\frac{2cx_{2}^{2}y_{2}}{1+\alpha x_{2}+\beta y_{2}+\alpha \beta x_{2}y_{2}}\\ & \leq \left(\sigma_{1}^{2}-2d_{1}-2p+1\right)x_{1}^{2}+\left(\sigma_{2}^{2}-2d_{2}+1\right)x_{2}^{2}+\left(\sigma_{3}^{2}-2d_{3}-2h+1\right)y_{1}^{2}\\ & \;+\left(\sigma_{4}^{2}-2d_{4}+1\right)y_{2}^{2}+2\left(a+p\right)x_{1}x_{2}+2hy_{1}y_{2}+\frac{2ecy_{1}}{\alpha \beta }-x_{1}^{2}-x_{2}^{2}-y_{1}^{2}-y_{2}^{2}. \end{array}$$

Let

(15) $$\begin{array}{cc} f(x_{1},x_{2},y_{1},y_{2})= & \left(\sigma_{1}^{2}-2d_{1}-2p+1\right)x_{1}^{2}+\left(\sigma_{2}^{2}-2d_{2}+1\right)x_{2}^{2}+\left(\sigma_{3}^{2}-2d_{3}-2h+1\right)y_{1}^{2}\\ & +\left(\sigma_{4}^{2}-2d_{4}+1\right)y_{2}^{2}+2\left(a+p\right)x_{1}x_{2}+2hy_{1}y_{2}+\frac{2ecy_{1}}{\alpha \beta }. \end{array}$$

Under Assumption 1, it is easy to find that function $f(x_{1},x_{2},y_{1},y_{2})$ has an upper bound. We assume that its upper bound is as follows

(16) $$\begin{array}{cc} M=\underset{\left(x_{1},x_{2},y_{1},y_{2}\right)\in R_{+}^{4}}{\sup} & \{f\left(x_{1},x_{2},y_{1},y_{2}\right)\}. \end{array}$$

Letting N=M+1 and noticing f(0,0,0,0)=0, we have N>0. According to Formula (14), we can obtain

(17) $$\begin{array}{cc} dV(x_{1},x_{2},y_{1},y_{2}) & \leq \left[N-\left(x_{1}^{2}+x_{2}^{2}+y_{1}^{2}+y_{2}^{2}\right)\right]dt+2\sigma_{1}x_{1}^{2}dB_{1}\left(t\right)\\ & \;+2\sigma_{2}x_{2}^{2}dB_{2}\left(t\right)+2\sigma_{3}y_{1}^{2}dB_{3}\left(t\right)+2\sigma_{4}y_{2}^{2}dB_{4}\left(t\right). \end{array}$$

By $It\hat{o}$’s formula, we have

(18) $$\begin{array}{cc} d[e^{t}V\left(x_{1},x_{2},y_{1},y_{2}\right)] & =e^{t}V\left(x_{1},x_{2},y_{1},y_{2}\right)dt+e^{t}dV\left(x_{1},x_{2},y_{1},y_{2}\right)\\ & \leq Ne^{t}dt+2\sigma_{1}x_{1}^{2}dB_{1}\left(t\right)+2\sigma_{2}x_{2}^{2}dB_{2}\left(t\right)+2\sigma_{3}y_{1}^{2}dB_{3}\left(t\right)+2\sigma_{4}y_{2}^{2}dB_{4}\left(t\right). \end{array}$$

Integrating both sides of Formula (18) from 0 to *t* and then taking expectations, we have

$$e^{t}E\left[V\left(x_{1},x_{2},y_{1},y_{2}\right)\right]\leq V\left(x_{1}\left(0\right),x_{2}\left(0\right),y_{1}\left(0\right),y_{2}\left(0\right)\right)+Ne^{t}-N.$$

Hence, we have

$$\underset{t\to \infty }{lim sup}E\left[V\left(X\left(t\right)\right)\right]\leq N,$$

where $X\left(t\right)=\left(x_{1},x_{2},y_{1},y_{2}\right)$. Then, we have

$$\underset{t\to \infty }{lim sup}{E[|X\left(t\right)|}^{2}]\leq N.$$

For any ϵ>0, let $H=\frac{\sqrt{N}}{\sqrt{\epsilon }}$. By Chebyshev’s inequality, we can obtain

$$P\left\{|X\left(t\right)|>H\right\}\leq \frac{E(|X\left(t\right){|}^{2})}{H^{2}}.$$

Then,

$$\underset{t\to \infty }{lim sup}P\left\{|X\left(t\right)|>H\right\}\leq \frac{N}{H^{2}}=\epsilon .$$

The proof of Theorem 2 is completed. □

## 5. Stochastic Extinction

In this section, we show that the population becomes extinct with probability one.

**Theorem** **3.** *Assume that $\left\{x_{1}\left(t\right),x_{2}\left(t\right),y_{1}\left(t\right),y_{2}\left(t\right)\right\}$ is the solution of System (3) with initial data $\{x_{1}\left(0\right),$$x_{2}\left(0\right),y_{1}\left(0\right),y_{2}\left(0\right)\}$. Then,*

*(i) all prey and predators die out exponentially with probability one, if $\left(2d_{1}+\sigma_{1}^{2}\right)\left(2a-2d_{2}-\sigma_{2}^{2}\right)<{\left(a-d_{1}-d_{2}\right)}^{2}$;*

*(ii) predators $y_{1}\left(t\right)$ and $y_{2}\left(t\right)$ die out exponentially with probability one, if $\left(2d_{3}+\sigma_{3}^{2}\right)\left(\frac{2ec}{\alpha }-2d_{4}-\sigma_{4}^{2}\right)<{\left(\frac{ec}{\alpha }-d_{3}-d_{4}\right)}^{2}$.*

**Proof of Theorem** **3.** According to System (3), we get

$$d\left(x_{1}+x_{2}\right)=dx_{1}+dx_{2}=L\left(x_{1}+x_{2}\right)dt+\sigma_{1}x_{1}dB_{1}\left(t\right)+\sigma_{2}x_{2}dB_{2}\left(t\right),$$

where $L\left(x_{1}+x_{2}\right)=ax_{2}-d_{1}x_{1}-b_{1}x_{2}^{2}-d_{2}x_{2}-\frac{cx_{2}y_{2}}{1+\alpha x_{2}+\beta y_{2}+\alpha \beta x_{2}y_{2}}$. Let $V\left(x_{1},x_{2}\right)=\ln\left(x_{1}+x_{2}\right)$. By $It\hat{o}$’s formula, we can obtain

(19) $$\begin{array}{cc} dV(x_{1},x_{2}) & =\frac{1}{x_{1}+x_{2}}\left(dx_{1}+dx_{2}\right)-\frac{1}{2{\left(x_{1}+x_{2}\right)}^{2}}\left[{\left(dx_{1}\right)}^{2}+{\left(dx_{2}\right)}^{2}\right],\\ & =[\frac{1}{x_{1}+x_{2}}\left(ax_{2}-d_{1}x_{1}-d_{2}x_{2}-b_{1}x_{2}^{2}-\frac{cx_{2}y_{2}}{1+\alpha x_{2}+\beta y_{2}+\alpha \beta x_{2}y_{2}}\right)\\ & \;-\frac{\sigma_{1}^{2}x_{1}^{2}}{2{\left(x_{1}+x_{2}\right)}^{2}}-\frac{\sigma_{2}^{2}x_{2}^{2}}{2{\left(x_{1}+x_{2}\right)}^{2}}]dt+\frac{\sigma_{1}x_{1}}{x_{1}+x_{2}}dB_{1}\left(t\right)+\frac{\sigma_{2}x_{2}}{x_{1}+x_{2}}dB_{2}\left(t\right). \end{array}$$

Then,

(20) $$\begin{array}{cc} LV(x_{1},x_{2}) & =\frac{1}{2{\left(x_{1}+x_{2}\right)}^{2}}[2\left(x_{1}+x_{2}\right)\left(ax_{2}-d_{1}x_{1}-d_{2}x_{2}-b_{1}x_{2}^{2}-\frac{cx_{2}y_{2}}{1+\alpha x_{2}+\beta y_{2}+\alpha \beta x_{2}y_{2}}\right)\\ & \;-\sigma_{1}^{2}x_{1}^{2}-\sigma_{2}^{2}x_{2}^{2}],\\ & \leq \frac{1}{2{\left(x_{1}+x_{2}\right)}^{2}}\left[2\left(x_{1}+x_{2}\right)\left(ax_{2}-d_{1}x_{1}-d_{2}x_{2}\right)-\sigma_{1}^{2}x_{1}^{2}-\sigma_{2}^{2}x_{2}^{2}\right]. \end{array}$$

We can write term

$$2\left(x_{1}+x_{2}\right)\left(ax_{2}-d_{1}x_{1}-d_{2}x_{2}\right)-\sigma_{1}^{2}x_{1}^{2}-\sigma_{2}^{2}x_{2}^{2},$$

in the following way:

$$\left(x_{1}\left(t\right),x_{2}\left(t\right)\right)\left[\begin{array}{cc} -2d_{1}-\sigma_{1}^{2} & a-d_{1}-d_{2}\\ a-d_{1}-d_{2} & 2a-2d_{2}-\sigma_{2}^{2} \end{array}\right]{\left(x_{1}\left(t\right),x_{2}\left(t\right)\right)}^{T}.$$

Letting the matrix

$$A_{1}=\left[\begin{array}{cc} -2d_{1}-\sigma_{1}^{2} & a-d_{1}-d_{2}\\ a-d_{1}-d_{2} & 2a-2d_{2}-\sigma_{2}^{2} \end{array}\right],$$

it is clear that matrix $A_{1}$ is negative definite under the condition in (i). Define $\lambda_{max}$ as the maximum eigenvalue of matrix $A_{1}$. According to the condition in (i), we obtain

$$\left(x_{1}\left(t\right),x_{2}\left(t\right)\right)\left[\begin{array}{cc} -2d_{1}-\sigma_{1}^{2} & a-d_{1}-d_{2}\\ a-d_{1}-d_{2} & 2a-2d_{2}-\sigma_{2}^{2} \end{array}\right]{\left(x_{1}\left(t\right),x_{2}\left(t\right)\right)}^{T}\leq -\left|\lambda_{max}\right|\left(x_{1}^{2}\left(t\right)+x_{2}^{2}\left(t\right)\right).$$

Therefore, we have

(21) $$\begin{array}{cc} dV\left(x_{1},x_{2}\right)=d\left(\ln\left(x_{1}+x_{2}\right)\right) & \leq \left[\frac{-|\lambda_{max}|\left(x_{1}^{2}+x_{2}^{2}\right)}{2{\left(x_{1}+x_{2}\right)}^{2}}\right]dt+\frac{\sigma_{1}x_{1}}{x_{1}+x_{2}}dB_{1}\left(t\right)+\frac{\sigma_{2}x_{2}}{x_{1}+x_{2}}dB_{2}\left(t\right),\\ & \leq -\frac{1}{4}\left|\lambda_{max}\right|dt+\frac{\sigma_{1}x_{1}}{x_{1}+x_{2}}dB_{1}\left(t\right)+\frac{\sigma_{2}x_{2}}{x_{1}+x_{2}}dB_{2}\left(t\right). \end{array}$$

Integrating both sides of Formula (19), it can be obtained that

(22) $$\begin{array}{c} \ln\left(x_{1}+x_{2}\right)\leq \ln\left(x_{1}\left(0\right)+x_{2}\left(0\right)\right)-\frac{1}{4}\left|\lambda_{max}\right|t+\int_{0}^{t}\frac{\sigma_{1}x_{1}}{x_{1}+x_{2}}dB_{1}\left(t\right)+\int_{0}^{t}\frac{\sigma_{2}x_{2}}{x_{1}+x_{2}}dB_{2}\left(t\right). \end{array}$$

Let $Z_{1}\left(t\right)=\int_{0}^{t}\frac{\sigma_{1}x_{1}}{x_{1}+x_{2}}dB_{1}\left(t\right)$ and $Z_{2}\left(t\right)=\int_{0}^{t}\frac{\sigma_{2}x_{2}}{x_{1}+x_{2}}dB_{2}\left(t\right)$, where $Z_{1}\left(t\right)$ and $Z_{2}\left(t\right)$ are local martingales. By the strong law of large numbers for local martingales (see, e.g., Reference [26]), we can obtain the following properties:

$$\lim_{t\to \infty }\frac{Z_{i}\left(t\right)}{t}=0\;\;a.s.\;\;i=1,2.$$

Therefore, we can get

$$\underset{t\to \infty }{lim sup}\frac{\ln(x_{1}+x_{2})}{t}\leq -\frac{1}{4}\left|\lambda_{max}\right|<0\;\;a.s.$$

Then, we have

$$\lim_{t\to \infty }x_{1}\left(t\right)\to 0,\;\;a.s.\;\;\lim_{t\to \infty }x_{2}\left(t\right)\to 0.\;\;a.s.$$

Then, with the extinction of the prey, we find the predator dies out according to System (3). The discussion of the predator population is similar. We have

(23) $$\begin{array}{cc} L(\ln\left(y_{1}+y_{2}\right)) & \leq \frac{1}{2{\left(y_{1}+y_{2}\right)}^{2}}\left[\left(y_{1}\left(t\right),y_{2}\left(t\right)\right)A_{2}{\left(y_{1}\left(t\right),y_{2}\left(t\right)\right)}^{T}\right], \end{array}$$

where

$$A_{2}=\left[\begin{array}{cc} -2d_{3}-\sigma_{3}^{2} & \frac{ec}{\alpha }-d_{3}-d_{4}\\ \frac{ec}{\alpha }-d_{3}-d_{4} & \frac{2ec}{\alpha }-2d_{4}-\sigma_{4}^{2} \end{array}\right].$$

Let ${\bar{\lambda }}_{max}$ be the maximum eigenvalue of matrix $A_{2}$. Under the condition in (ii), we have

(24) $$\begin{array}{c} \ln\left(y_{1}+y_{2}\right)\leq \ln\left(y_{1}\left(0\right)+y_{2}\left(0\right)\right)-\frac{1}{4}\left|{\bar{\lambda }}_{max}\right|t+\int_{0}^{t}\frac{\sigma_{3}y_{1}}{y_{1}+y_{2}}dB_{3}\left(t\right)+\int_{0}^{t}\frac{\sigma_{4}y_{2}}{y_{1}+y_{2}}dB_{4}\left(t\right). \end{array}$$

Then, it can be obtained that

$$\underset{t\to \infty }{lim sup}\frac{\ln(y_{1}+y_{2})}{t}\leq -\frac{1}{4}\left|{\bar{\lambda }}_{max}\right|<0\;\;a.s.$$

which implies

$$\lim_{t\to \infty }y_{1}\left(t\right)\to 0,\;\;a.s.\;\;\lim_{t\to \infty }y_{2}\left(t\right)\to 0.\;\;a.s.$$

The proof of Theorem 3 is completed. □

## 6. Numerical Simulations

In this section, we illustrate our theoretical results using the numerical simulations of System (3). We randomly selected the initial condition in (0,1). The initial state of the system is (0.6324,0.8147,0.127,0.2785). We used the Milstein method mentioned in Reference [29] to substantiate the analytical findings. Consider the discretization transformation of System (3):

(25) $$\begin{array}{c} \left\{\begin{array}{c} x_{1}^{j+1}=x_{1}^{j}+\left(ax_{2}^{j}-d_{1}x_{1}^{j}-fx_{1}^{j}\right)\Delta t+\sigma_{1}x_{1}^{j}\sqrt{\Delta t}\epsilon_{1,j}+\frac{1}{2}\sigma_{1}^{2}x_{1}^{j}\left(\epsilon_{1,j}^{2}\Delta t-\Delta t\right),\\ x_{2}^{j+1}=x_{2}^{j}+\left(fx_{1}^{j}-d_{2}x_{2}^{j}-b_{1}x_{2}^{2j}-\frac{cx_{2}^{j}y_{2}^{j}}{1+\alpha x_{2}^{j}+\beta y_{2}^{j}+\alpha \beta x_{2}^{j}y_{2}^{j}}\right)\Delta t+\sigma_{2}x_{2}^{j}\sqrt{\Delta t}\epsilon_{2,j}+\frac{1}{2}\sigma_{2}^{2}x_{2}^{j}\left(\epsilon_{2,j}^{2}\Delta t-\Delta t\right),\\ y_{1}^{j+1}=y_{1}^{j}+\left(\frac{ecx_{2}^{j}y_{2}^{j}}{1+\alpha x_{2}^{j}+\beta y_{2}^{j}+\alpha \beta x_{2}^{j}y_{2}^{j}}-d_{3}y_{1}^{j}-hy_{1}^{j}\right)\Delta t+\sigma_{3}y_{1}^{j}\sqrt{\Delta t}\epsilon_{3,j}+\frac{1}{2}\sigma_{3}^{2}y_{1}^{j}\left(\epsilon_{3,j}^{2}\Delta t-\Delta t\right),\\ y_{2}^{j+1}=y_{2}^{j}+\left(hy_{1}^{j}-d_{4}y_{2}^{j}-b_{2}y_{2}^{2j}\right)\Delta t+\sigma_{4}y_{2}^{j}\sqrt{\Delta t}\epsilon_{4,j}+\frac{1}{2}\sigma_{4}^{2}y_{2}^{j}\left(\epsilon_{4,j}^{2}\Delta t-\Delta t\right), \end{array}\right. \end{array}$$

where time increment Δt is positive and $\epsilon_{i,j}$(i=1,2,3,4) are the Gaussian random variables that follow distribution N(0,1). For System (3), the parameters are selected as follows: 

(i). a=0.7, $b_{1}=0.9$, $b_{2}=0.9$, c=0.8, $e=\frac{7}{8}$, p=0.3, h=0.5, α=0.8, β=0.5, $d_{1}=0.9$, $d_{2}=0.5$, $d_{3}=0.5$, $d_{4}=0.5$, $\sigma_{1}^{2}=0.5$, $\sigma_{2}^{2}=1$, $\sigma_{3}^{2}=0.1$, $\sigma_{4}^{2}=0.8$.

(ii). a=0.3, $b_{1}=0.2$, $b_{2}=0.8$, c=0.7, e=0.7, p=0.6, h=0.5, α=0.8, β=0.6, $d_{1}=0.2$, $d_{2}=0.1$, $d_{3}=0.7$, $d_{4}=0.7$, $\sigma_{1}^{2}=0.01$, $\sigma_{2}^{2}=0.01$, $\sigma_{3}^{2}=0.2$, $\sigma_{4}^{2}=0.5$.

**Figure 1:**
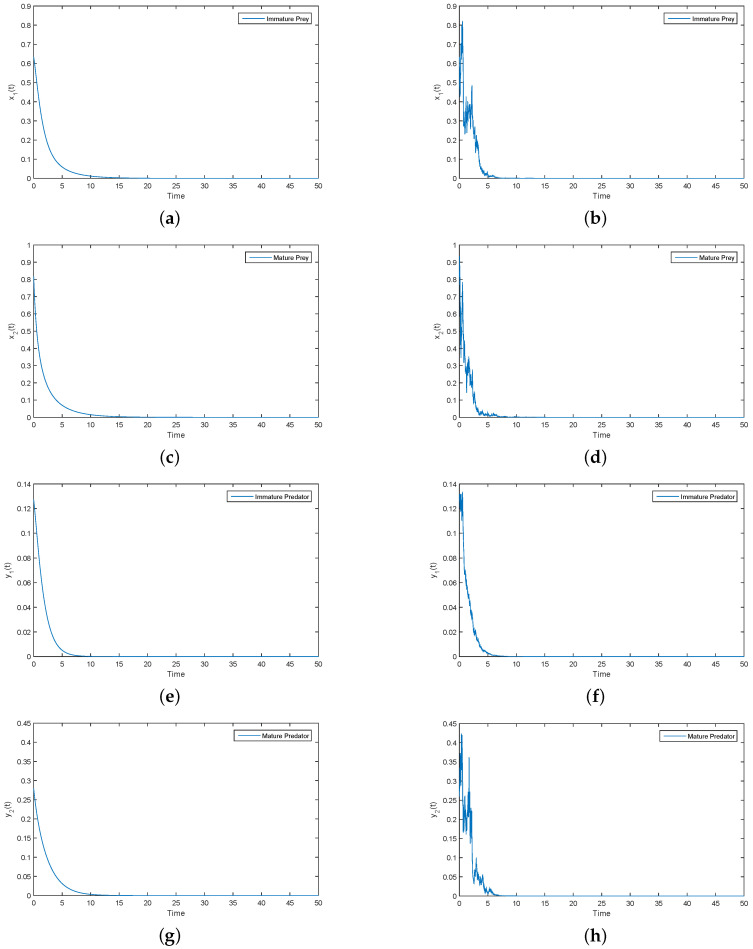
(**a**,**c**,**e**,**g**) Solutions of $x_{1}\left(t\right)$, $x_{2}\left(t\right)$, $y_{1}\left(t\right)$ and $y_{2}\left(t\right)$ for deterministic System (2), respectively; (**b**,**d**,**f**,**h**) solutions of $x_{1}\left(t\right)$, $x_{2}\left(t\right)$, $y_{1}\left(t\right)$ and $y_{2}\left(t\right)$ for perturbation System (3), respectively.

It is easy to verify that Parameters (i) satisfy the condition of the extinction of the prey population in Theorem 3. The corresponding numerical results are shown in Figure 1.

As can be clearly seen from Figure 1, $x_{1}\left(t\right)$, $x_{2}\left(t\right)$, $y_{1}\left(t\right)$ and $y_{2}\left(t\right)$ tend to zero in both the deterministic and stochastic models. Under Parameter (i), we have $\left(2d_{1}+\sigma_{1}^{2}\right)\left(2a-2d_{2}-\sigma_{2}^{2}\right)<{\left(a-d_{1}-d_{2}\right)}^{2}$. By Theorem 3, $x_{1}\left(t\right)$, $x_{2}\left(t\right)$, $y_{1}\left(t\right)$ and $y_{2}\left(t\right)$ tend to become extinct. Numerical simulations clearly support this result (see Figure 1). Therefore, Figure 1 provides evidence for the accuracy of Conclusion (i) in Theorem 3. Then, Under Parameter (ii), the corresponding numerical simulation results are as follows.

**Figure 2:**
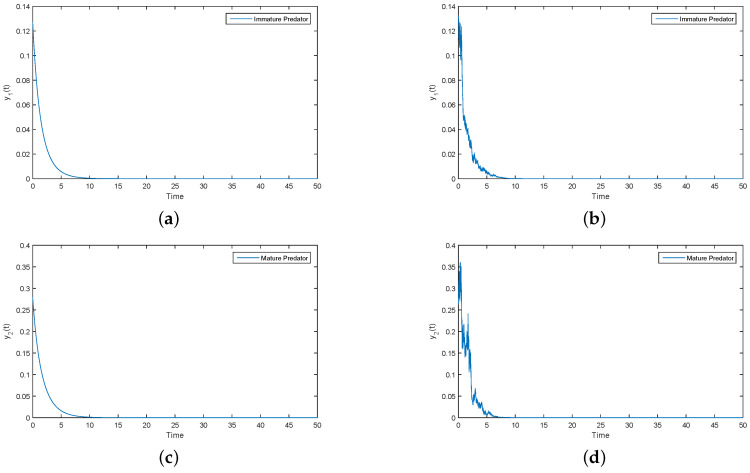
(**a**,**c**) Solutions of immature and mature predator population for deterministic System (2), respectively; (**b**,**d**) solutions of immature and mature predator of perturbation System (3), respectively.

**Figure 3:**
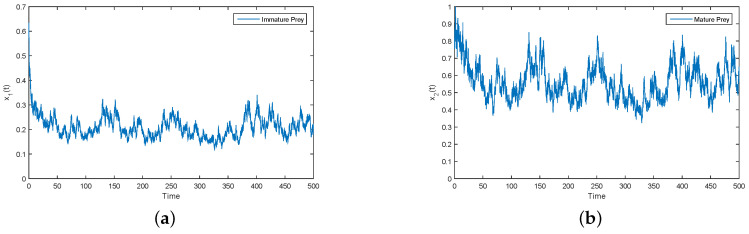
(**a**,**b**) Solutions of $x_{1}\left(t\right)$ and $x_{2}\left(t\right)$ for perturbation System (3), respectively.

As can be clearly seen from Figure 2, $y_{1}\left(t\right)$ and $y_{2}\left(t\right)$ tend to zero in both the deterministic and stochastic models. By calculation, we can find that Parameter (ii) satisfies condition $\left(2d_{3}+\sigma_{3}^{2}\right)\left(\frac{2ec}{\alpha }-2d_{4}-\sigma_{4}^{2}\right)<{\left(\frac{ec}{\alpha }-d_{3}-d_{4}\right)}^{2}$. According to Theorem 3, $y_{1}\left(t\right)$ and $y_{2}\left(t\right)$ tend to become extinct. Numerical simulations clearly support this result (see Figure 2). Meanwhile, under Parameter (ii), we give the trajectories of $x_{1}\left(t\right)$ and $x_{2}\left(t\right)$ over a long period of time (see Figure 3). Figure 3 shows that the immature prey and mature prey are permanence for a long time.

Under the condition of Parameter (ii), according to Figure 2 and Figure 3, the predator tends to become extinct and the prey survives for a long time. In nature, this situation is reasonable.

## 7. Conclusions

In this paper, we researched the predator–prey system with a Crowley–Martin functional response function and environmental noise. In Reference [5], we found that the predator-dependent functional response is more reasonable than the prey-dependent functional response. In particular, the Crowley–Martin functional response is more suitable for the case that the predator feeding rate is decreased by higher predator density. Compared with Holling Types I–III functional responses, the Crowley–Martin functional response has more complex forms. From an analysis point of view, the theoretical analysis of predator–prey system with a Crowley–Martin functional response is more difficult, and the results are more complex. Meanwhile, we know that the system is inevitably affected by environmental noise. Therefore, we researched the predator–prey model with a Crowley–Martin functional response function and environmental noise. On this basis, we first attempted to consider the stage structure on both prey and predator. First, we proved the existence and uniqueness of the global positive solution of System (3). Next, we pointed out that the positive solution is stochastically bounded. Then, we gave sufficient conditions for the extinction of the predator and prey populations in two cases. Some interesting questions deserve further investigation; we will research the stability and stationary distribution of System (3) (see Reference [30]), and consider the impact of sudden changes and time delays on population characteristics (see Reference [31]) in the future. In addition, we will research the chaotic behavior of a predator–prey system and the Allee effect (see References [32,33]).
